# Effect of Anthocyanin-Rich Extract of Sour Cherry for Hyperglycemia-Induced Inflammatory Response and Impaired Endothelium-Dependent Vasodilation

**DOI:** 10.3390/nu12113373

**Published:** 2020-11-02

**Authors:** Arnold Markovics, Attila Biró, Andrea Kun-Nemes, Mónika Éva Fazekas, Anna Anita Rácz, Melinda Paholcsek, János Lukács, László Stündl, Judit Remenyik

**Affiliations:** 1 Institute of Food Technology, University of Debrecen, H-4032 Debrecen, Hungary; arnoldmarkovich@gmail.com (A.M.); biro.attila@agr.unideb.hu (A.B.); andrea.nemes83@gmail.com (A.K.-N.); fazekas.monika@agr.unideb.hu (M.É.F.); stundl@agr.unideb.hu (L.S.); 2 Department of Human Genetics, University of Debrecen, H-4032 Debrecen, Hungary; racz.anna@med.unideb.hu (A.A.R.); paholcsek.melinda@med.unideb.hu (M.P.); 3 Department of Obstetrics and Gynaecology, University of Debrecen, H-4032 Debrecen, Hungary; lukacs.janos@med.unideb.hu

**Keywords:** hyperglycemia, anthocyanins, endothelial dysfunction, vasodilation

## Abstract

Diabetes mellitus (DM)-related morbidity and mortality are steadily rising worldwide, affecting about half a billion people worldwide. A significant proportion of diabetic cases are in the elderly, which is concerning given the increasing aging population. Proper nutrition is an important component in the effective management of diabetes in the elderly. A plethora of active substances of plant origin exhibit potency to target the pathogenesis of diabetes mellitus. The nutraceutical and pharmaceutical effects of anthocyanins have been extensively studied. In this study, the effect of Hungarian sour cherry, which is rich in anthocyanins, on hyperglycemia-induced endothelial dysfunction was tested using human umbilical cord vein endothelial cells (HUVECs). HUVECs were maintained under both normoglycemic (5 mM) and hyperglycemic (30 mM) conditions with or without two concentrations (1.50 ng/µL) of anthocyanin-rich sour cherry extract. Hyperglycemia-induced oxidative stress and inflammatory response and damaged vasorelaxation processes were investigated by evaluating the level of reactive oxygen species (ROS) and gene expression of four proinflammatory cytokines, namely, tumor necrosis factor-alpha (TNF-α), interleukin-6 (IL-6), interleukin-8 (IL-8), and interleukin-1α (IL-1α), as well as the gene expression of nitric oxide synthase (NOS) endothelin-1 (ET-1) and endothelin-converting enzyme-1 (ECE-1). It was found that hyperglycemia-induced oxidative stress was significantly suppressed by anthocyanin-rich sour cherry extract in a concentration-dependent manner. The gene expression of the tested proinflammatory cytokines increased under hyperglycemic conditions but was significantly reduced by both 1 and 50 ng/µL anthocyanin-rich sour cherry extract. Further, although increased ET-1 and ECE-1 expression due to hyperglycemia was reduced by anthocyanin-rich sour cherry extract, NOS expression was increased by the extract. Collectively, these data suggest that anthocyanin-rich sour cherry extract could alleviate hyperglycemia-induced endothelial dysfunction due to its antioxidant, anti-inflammatory, and vasorelaxant effects.

## 1. Introduction

Life expectancy has increased as a result of novel scientific and technological advances and a decline in poverty, which has facilitated a reduction of communicable diseases [1]. This has shifted the attention of the medical community to treatment of noncommunicable, or chronic, conditions, such as diabetes mellitus (DM). Diabetes mellitus is one of the most common disorders in older adults. In old age (≥60–65 years), DM exhibits higher prevalence than in younger people, posing a serious public health problem in both developed and developing countries [2]. 

Diabetes mellitus is a condition characterized by persistent hyperglycemia in the blood. Cases of diabetes can be largely classified into two types. In type 1 diabetes (T1D), the cause is an absolute deficiency of insulin secretion due to an autoimmune-mediated response. Type 2 diabetes (T2D), which is much more prevalent in the elderly, is caused by a combination of resistance to insulin action and insufficient compensatory insulin secretory response [3]. Although the progression of diabetes and the emergence of complications have been extensively studied, the molecular mechanisms have not yet been fully elucidated [4]. Nevertheless, there is common agreement that microvascular damage is a key early event in the development of many diabetic complications [5]. The microvascular endothelium is thought to be a major target of hyperglycemic damage as endothelial cells take up glucose passively in an insulin-independent manner and cannot downregulate the glucose transport rate when glucose concentration is elevated, resulting in intracellular hyperglycemia, which significantly affects endothelial cell biology [5].

Hyperglycemia causes tissue damage through four major mechanisms: increased intracellular formation of advanced glycation end products (AGEs), activation of protein kinase C (PKC) isoforms, increased flux of polyol, and via the hexosamine pathway. The collective intensification of these pathways results in a single upstream event: mitochondrial overproduction of reactive oxygen species (ROS) [6]. This consequence of intracellular hyperglycemia contributes to degeneration of microvasculature and leads to progression of diabetic complications [5].

The close relationship between oxidative stress and inflammation has been extensively studied and well documented [7]. Persistent hyperglycemia triggers expression of various proinflammatory cytokines and chemokines, including interleukin-6 (IL-6), interleukin-8 (IL-8), interleukin-1α (IL-1α), and tumor necrosis factor-alpha (TNF-α, which in turn leads to an increase in the inflammatory response. Hyperglycemia-induced inflammation, in synergy with oxidative stress, plays a key role in the pathogenesis of vascular complication of diabetes [8].

The endothelial dysfunction caused by hyperglycemia and consequent inflammation as well as oxidative stress is characterized by impaired endothelium-dependent vasodilation. Several studies have demonstrated that an imbalance of mediators of endothelium-dependent vasodilation is eventuated in hyperglycemic conditions [9]. Amongst the major factors that regulate vasodilation are nitric oxide (NO) and endothelin (ET)-1 [10]. NO is a potent vasodilator produced by nitric oxide synthase (NOS) from the amino acid precursor L-arginine. ET-1 is a locally acting vasoconstrictor produced in endothelial cells by endothelin-converting enzyme (ECE)-1 [11]. Several studies have demonstrated that NO-mediated vasodilation is abnormal in patients with T2D [12]. Increased circulating levels of ET-1 have been found in patients with diabetes [13]. NOS is downregulated while endothelin-1 expression is increased in hyperglycemic conditions [14].

Epidemiological studies have indicated an inverse association between fruit and vegetable intake and the risk of DM. Several dietary patterns consisting of combinations of different foods or food groups are beneficial for diabetes management [15]. Proper nutrition and diet are important factors in the prevention and management of DM. A recent study highlighted the effect of anthocyanin-rich foods or extracts on vascular function in adults [16].

Anthocyanins are nutrients that belong to polyphenols and are mainly found in dark fruits and vegetables [17]. Anthocyanins are polyhydroxy or polymethoxy derivatives of 2-phenylbenzophyryllium in terms of their chemical structure. A phenolic compound consists of two aromatic rings (A and B rings) linked by a three-carbon chain that forms an oxygenated heterocyclic ring (C ring) [18]. Anthocyanins are able to exert, inter alia, antidiabetic effects. In vitro, in vivo, and a few clinical studies have suggested that dietary anthocyanins could ameliorate insulin resistance and offer health benefits in diabetic conditions [19,20,21].

Interestingly, the molecular mechanisms mentioned above, including excessive ROS production, increased expression of proinflammatory cytokines, and deterioration of vasorelaxant function, can have severe consequences in old age, regardless of diabetes. Vascular aging is a key process affecting the health status of the aged population [22].

In this study, the effect of anthocyanin-rich sour cherry extract was investigated on hyperglycemia-induced inflammatory response, oxidative stress, and impaired endothelium-dependent vasodilation using a culture of human umbilical vein endothelial cells (HUVECs) in order to obtain information on the possible beneficial effect of anthocyanin-rich sour cherry extract. The results may highlight the importance of a diversified diet for healthy aging, especially with regard to cherry consumption.

## 2. Material and Methods 

### 2.1. Materials

Purified anthocyanin-rich sour cherry extract was prepared by solid-phase extraction procedure following an established protocol [23]. Glucose was purchased from Biosera (Biosera, Nuaille, France).

### 2.2. Methods

#### 2.2.1. Isolation and Cell Culturing

The HUVECs originated from human umbilical cords that were collected from normal-term placenta and obtained from the Department of Obstetrics and Gynecology, Clinical Centre, University of Debrecen, Debrecen, Hungary. HUVECs were maintained according to the method previously described [24]. Cells were cultured in M199 medium (Biosera, Nuaille, France) supplemented with 10% (*v*/*v*) fetal bovine serum (Biosera, Nuaille, France), 10% (*v*/*v*) endothelial cell growth (EGM)-2 complex medium (Lonza, Basel, Switzerland), 1.2% (*v*/*v*) 2 mM glutamine (1:500; Biosera, Nuaille, France) 1.2% (*v*/*v*) 1X penicillin/streptomycin (Biosera, Nuaille, France), 1.2% (*v*/*v*) 1X penicillin/streptomycin (Biosera, Nuaille, France), and 1% amphotericin B. Cells were subcultured at 80–100% confluence and incubated at 37 °C with 5% CO_2_ level.

#### 2.2.2. Determination of Cellular Viability

##### MTT Assay

The viability of HUVECs was determined by 3-(4,5-dimethylthiazol-2-yl)-2,5 diphenyltetrazolium bromide (MTT) assay (Duchefa Biochemie, Haarlem, the Netherlands). Cells were plated in 96-well plates (15,000 cells/well) in quadruplicate and treated as indicated. Cells were then incubated with 0.5 mg/mL MTT reagent for 3 h. The formazan crystals were dissolved in 100 µL solubilizing solution (81% (*v*/*v*) isopropyl alcohol (Serva, Heidelberg, Germany), 9% (*v*/*v*) 1 M HCl (Serva, Heidelberg, Germany), and 10% (*v*/*v*) Triton X-100 (Serva, Heidelberg, Germany)) and determined colorimetrically at 465 nm using a Clariostar microplate reader (BMG Labtech, Ortenberg, Germany). The results are expressed as a percentage of vehicle control, regarded as 100%.

##### Nile Red Assay

For quantitative measurement of polar lipid content of HUVECs, 1 µg/mL Nile Red (Sigma-Aldrich, St. Louis, MO, USA) was used. Cells were cultured in black 96-well plates (15,000 cells/well) in quadruplicate and treated as indicated. The plates were then incubated at 37 °C for 30 min, and fluorescence was measured (485 nm excitation and 565 nm emission wavelengths) using a Clariostar microplate reader (BMG Labtech, Ortenberg, Germany). The results are expressed as a percentage of vehicle control, regarded as 100%.

##### Determination of Apoptosis

One of the earliest markers of apoptosis is the decrease in mitochondrial membrane potential. The membrane potential of HUVECs was determined using DilC_1_(5) fluorescence dye (ENZO, Farmingdale, NY, USA). Cells were seeded into 96-well plates (15,000 cells/well) in quadruplicate and treated as indicated. After removal of the medium, cells were incubated for 30 min with DilC_1_(5) solution and then washed with PBS. The fluorescence intensity of DilC_1_(5) was measured at 630 nm excitation and 670 nm emission wavelengths using a Clariostar microplate reader (BMG Labtech, Ortenberg, Germany). The results are expressed as a percentage of vehicle control, regarded as 100%.

##### Determination of Necrosis

Necrotic processes were determined by SYTOX Green staining (Thermo Fisher Scientific, Waltham, MA, USA). Cells were plated into 96-well plates (15,000 cells/well) in quadruplicate and treated as indicated. After the removal of medium, HUVECs were incubated for 30 min with 1 µM SYTOX Green solution. Following incubation, cells were washed with phosphate-buffered saline (PBS), and the fluorescence intensity of SYTOX Green was measured at 490 nm excitation and 520 nm emission wavelengths using a Clariostar microplate reader (BMG Labtech, Ortenberg, Germany). The results are expressed as a percentage of vehicle control, regarded as 100%.

##### Determination of Level of ROS

Production of ROS by HUVECs was determined by 2′,7′-dichlorofluorescin diacetate (DCFDA) staining (Sigma-Aldrich, St. Louis, MO, USA). Cells were seeded into 24-well plates (100,000 cells/well) in quadruplicate. To label intracellular ROS, cells were incubated with 100 µM DCFDA solution. After 1 h of incubation, cells were washed with PBS and treated as indicated. The fluorescence intensity (excitation = 485 nm; emission = 530 nm) of DCFDA was measured using a Clariostar microplate reader (BMG Labtech, Ortenberg, Germany). The results are expressed as a percentage of vehicle control, regarded as 100%.

##### Gene Expression Studies by qPCR 

qPCR was performed on a Roche LightCycler 480 System (Roche, Basel, Switzerland) using the 5′ nuclease assay. Total RNA was isolated using Extrazole (Blirt, Gdansk, Poland). One microgram of total RNA was reverse transcribed into cDNA using a LunaScript RT SuperMix kit (PCR Biosystems, London, UK). Amplification was performed using the Luna Universal Probe qPCR Master Mix (PCR Biosystems, London, UK). Glyceraldehyde-3-phosphate dehydrogenase (GAPDH) was determined as internal control. The results are expressed relative to 100% for the control group.

##### Statistical Analysis

Data were analyzed and presented by GraphPad Prism 8.3.1 (GraphPad Software, La Jolla, CA, USA). For multiple comparisons, results were analyzed by ANOVA followed by modified *t*-test for repeated measures according to Bonferroni’s method. The data are presented as mean ± SEM) (in the case of PCR analysis, results are presented as mean ± SD).

### 2.3. Ethics

The study was conducted in accordance with the Declaration of Helsinki, and the protocol was approved by the ethics committee of the University of Debrecen (registration number RKEB/IKEB 3712-2012).

## 3. Results

### 3.1. Preliminarily Experiments 

The main anthocyanin components of sour cherry are cyanidin-3-rutinoside, cyanidin-3-o-glucoside, and cyanidin-3-o-glucosil-rutinoside based on our preliminarily chromatographic analysis [23].

Isolated HUVECs were characterized to positive and negative marker expression using flow cytometry, a method that was routinely used in our recent HUVEC-oriented studies [25].

The optimal concentration of anthocyanin-rich sour cherry extract was decided based on MTT, apoptosis, and necrosis assays [25].

### 3.2. Effect of Anthocyanin-Rich Sour Cherry Extract on the Viability of HUVECs Maintained in a Hyperglycemic State

**Figure 1:**
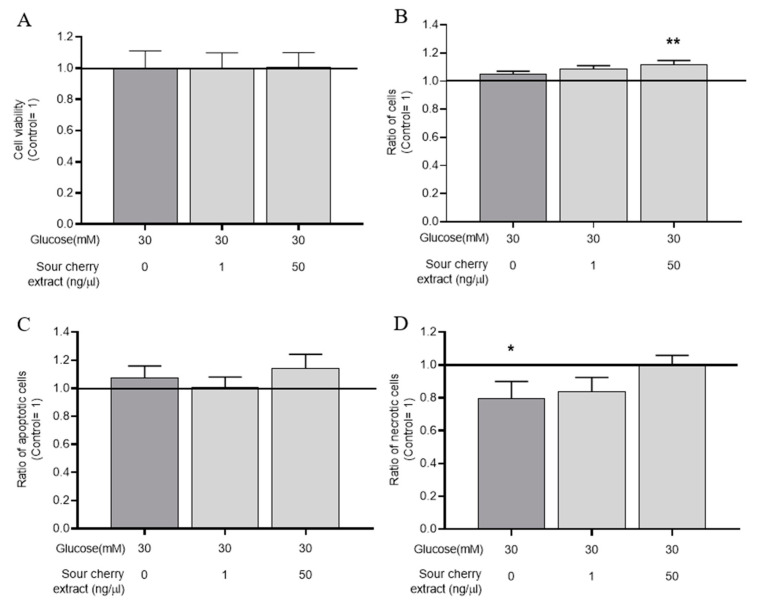
Combined use of anthocyanin-rich sour cherry extract and high glucose does not influence viability of human umbilical cord vein endothelial cells (HUVECs). Viability of HUVECs was monitored following 48 h treatment by 3-(4,5-dimethylthiazol-2-yl)-2,5 diphenyltetrazolium bromide (MTT) (**A**) and Nile Red (**B**) assays. Early necrotic and apoptotic processes of HUVECs were monitored following 48 h treatment by DilC1(5) (**C**) and SYTOX Green (**D**) assays. Results are expressed as percentage of untreated control (100%, solid line), with normoglycemia (5 mM) as mean ± SEM of four independent determinations. * and ** mark significant (*p* < 0.05 and *p* < 0.01, respectively) differences compared to the vehicle control group.

Based on the optimal anthocyanin-rich sour cherry extract concentration determined as described above and in a recent study [26] discussing the absorption of anthocyanins and flavanones in humans, we selected anthocyanin-rich sour cherry extract at 1 and 50 ng/µL for further experiments. To investigate the combined effect of the hyperglycemic environment and anthocyanin-rich sour cherry extract, HUVECs were maintained at high glucose levels and treated with anthocyanin-rich sour cherry extract as indicated. Our experiments with MTT assay showed that the combined use of anthocyanin-rich sour cherry extract and high glucose concentration did not significantly reduce cell viability, even after 48 h treatment (Figure 1A). This was also tested using the Nile Red assay, and the same result was observed. While MTT determines cell viability based on mitochondrial dehydrogenase activity, the fluorescent dye Nile Red determines the relative cell number based on the polar lipid content [27]. The results (Figure 1B) of Nile Red assay showed that the combined use of anthocyanin-rich sour cherry extract and high glucose concentration, in line with the MTT data, did not evoke change in cell viability.

Our results required further verification as the onset of early apoptotic and necrotic processes was certainly not detectable in the experiments used above. For this purpose, we examined early apoptotic processes by DilC_1_(5) assay (Figure 1C) and early necrotic processes by SYTOX Green labeling (Figure 1D). Although the initiation of a small necrotic process was observed with high glucose treatment, anthocyanin-rich sour cherry extract was able to prevent this and, when used in combination with high glucose, did not cause either necrosis or apoptosis in our delayed conditions on HUVECs.

These results showed that the combined use of anthocyanin-rich sour cherry extract and high glucose concentration had no cytotoxic effect.

### 3.3. Anthocyanin-Rich Sour Cherry Extract Exerts a Potent Antioxidant Effect

**Figure 2:**
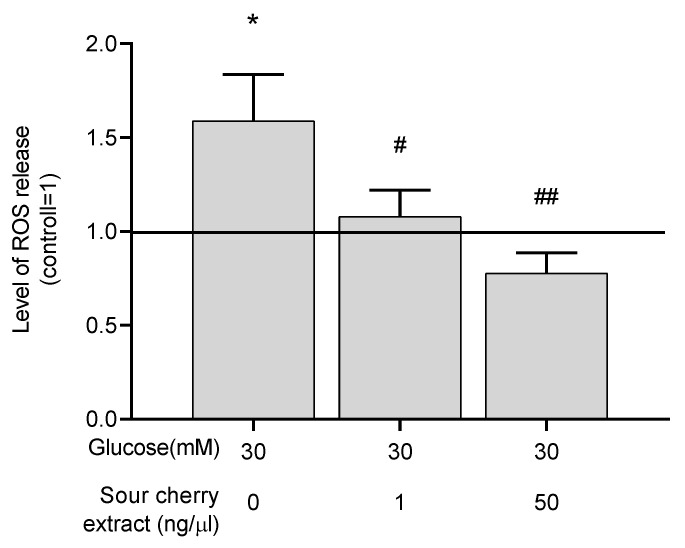
Anthocyanin-rich sour cherry extract decreases production of reactive oxygen species (ROS) under hyperglycemic condition. Production of ROS by HUVECs was monitored following 48 h treatment by 2′,7′-dichlorofluorescin diacetate (DCFDA) staining. Results are expressed as the percentage of untreated control (100%, solid line) with normoglycemia (5 mM) as mean ± SEM of four independent determinations. * marks a statistically significant difference (*p* < 0.05) compared to the vehicle control group. ^#^, ^##^ marks significant (*p* < 0.05, *p* < 0.001) differences as indicated compared to the untreated control with hyperglycemia (30 mM).

A study has reported the strong antioxidant effect of anthocyanins [28]. We intended to investigate the potential antioxidant effect of anthocyanin-rich sour cherry extract. To assess the antioxidant capacity of anthocyanin-rich sour cherry extract, the level of ROS was measured in hyperglycemic conditions with or without the extract. As expected, high glucose concentrations resulted in elevated ROS levels in our HUVECs model. Anthocyanin-rich sour cherry extract was able to eliminate this increment (Figure 2), indicating the potent antioxidant effect of the extract.

### 3.4. Anthocyanin-Rich Sour Cherry Extract Reduces Gene Expression of Proinflammatory Cytokines

**Figure 3:**
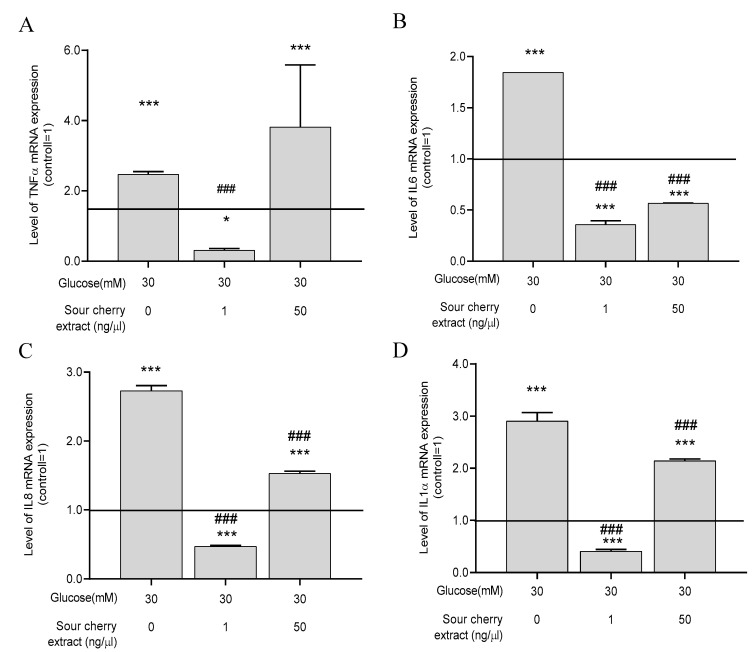
The anti-inflammatory action of anthocyanin-rich sour cherry extract. qPCR analyses of gene expression of tumor necrosis factor-alpha (TNF-α) (**A**), interleukin-6 (IL-6) (**B**), interleukin-8 (IL-8) (**C**), and interleukin-1α (IL-1α) (**D**) on HUVECs following the indicated seven days of simultaneous treatment. Data are presented using the ΔΔCT method regarding glyceraldehyde-3-phosphate dehydrogenase (GAPDH)-normalized mRNA expressions of the untreated control (100%, solid line) with normoglycemia (5 mM) mean ± SD of two independent determinations. * and *** mark significant (*p* < 0.05 and *p* < 0.001, respectively) differences as indicated compared to the untreated control with normoglycemia (5 mM). ^###^ marks significant (*p* < 0.001) differences as indicated compared to the untreated control with hyperglycemia (30 mM).

Numerous studies have shown a strong association between persistent hyperglycemia and inflammation. Increased secretion of proinflammatory cytokines is a manifestation of hyperglycemia-induced inflammatory processes [7]. In order to obtain insight into the inflammatory processes induced by hyperglycemia, we examined the expression levels of four proinflammatory cytokines, namely, TNF-α, IL-6, IL-8, and IL-1α, under hyperglycemic conditions with or without anthocyanin-rich sour cherry extract. First, these genes were examined after 48 h of incubation, when ROS production was the most obvious in our experimental setup. No significant change in the expression of proinflammatory cytokines was observed at this sampling time. Therefore, we assessed whether a differential expression occurred long-term by evaluating the expression after seven days. After seven days, high glucose concentration significantly increased the expression of the previously mentioned proinflammatory cytokines compared to the untreated control maintained at normal glucose levels (Figure 3). Anthocyanin-rich sour cherry extract was able to significantly suppress this effect at both 1 and 50 ng/µL concentrations, except for the case of TNF-α (Figure 3A), which was decreased significantly by 1 ng/µL concentration of anthocyanin-rich sour cherry extract.

### 3.5. Anthocyanin-Rich Sour Cherry Extract Enhances Expression of NOS and Decreases Expression of ET-1 and ECE-1

**Figure 4:**
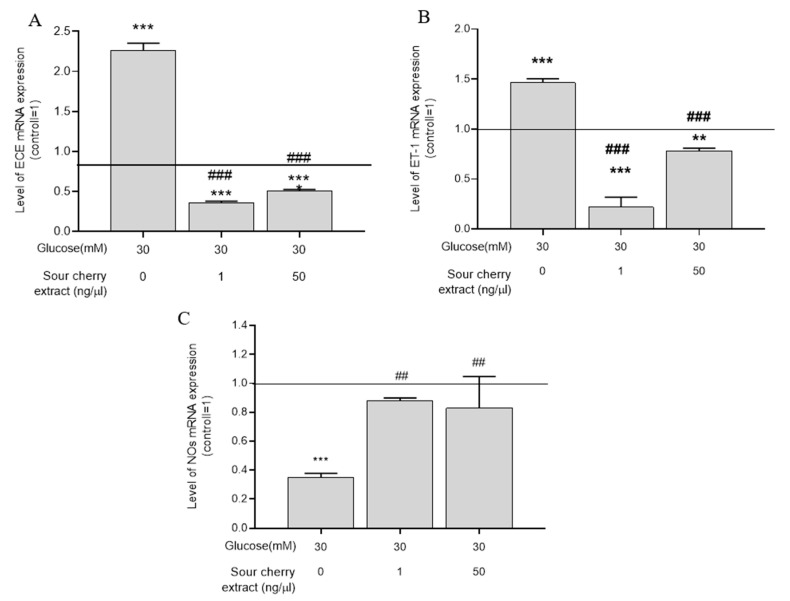
Vasorelaxant effect of anthocyanin-rich sour cherry extract. qPCR analyses of gene expression of endothelin-converting enzyme-1 (ECE-1) (**A**), endothelin-1 (ET-1) (**B**), and nitric oxide synthase (NOS) (**C**) on HUVECs following the indicated seven days of simultaneous treatment. Data are presented using the ΔΔCT method regarding GAPDH-normalized mRNA expressions of the untreated control (100%, solid line) with normoglycemia (5 mm) mean ± SD of two independent determinations. ** and *** mark significant (*p* < 0.01 and *p* < 0.001, respectively) differences as indicated compared to the untreated control with normoglycemia (5 mM). ^##^ and ^###^ mark significant (*p* < 0.01 and *p* < 0.001, respectively) differences as indicated compared to the untreated control with hyperglycemia (30 mM).

As a result of prolonged and direct contact with the hyperglycemic milieu, the vasorelaxation process is impaired [29]. Deficiency in bioavailable NO is one of the major features of hyperglycemia-induced endothelial dysfunction [30]. Moreover, the biosynthesis of certain vasoconstrictors, such as ET-1, is increased. Given the increase in the inflammatory response of cells exposed to persistent hyperglycemia (seven days), we investigated the expression levels of proteins that play a key role in endothelial cell vasorelaxation processes. Because a substantial inflammatory effect could only be elicited following seven days of treatment, the effect of high glucose levels on vasorelaxation was examined at this time. HUVECs treated at a concentration of 30 mM glucose significantly increased the gene expression of ET-1 (Figure 4A) as well as the expression of ECE-1 (Figure 4B), which produces its active form. Anthocyanin-rich sour cherry extract was able to significantly reduce the hyperglycemia-induced enhanced gene expression of ET-1 (Figure 4A) and ECE-1 (Figure 4B).

In parallel, the expression of NOS, which is responsible for vasodilation, was significantly decreased. Anthocyanin-rich sour cherry extract was able to increase the gene expression of NOS. 

## 4. Discussion

In our current experimental design, hyperglycemia-induced endothelial dysfunction was investigated. Before evaluating hyperglycemia-induced endothelial changes, we examined the combined effect of the hyperglycemic environment and anthocyanin-rich cherry extract. To monitor the negative effects of possible cross reactions, the viability of HUVECs was assessed. The combined use of anthocyanin-rich cherry extract and high glucose concentration did not show a cytotoxic effect, even after 48 h. We further examined hyperglycemia-induced endothelial changes by evaluating inflammatory response, oxidative stress, and damaged endothelium-dependent vasodilation. Anthocyanin-rich sour cherry extract was able to eliminate, in a concentration-dependent manner, hyperglycemia-induced ROS production in HUVECs, thereby alleviating oxidative stress. The investigated extract also alleviated hyperglycemia-induced increased expression of proinflammatory cytokines, indicating its immunomodulatory effects. Furthermore, hyperglycemia-induced impaired vasodilation processes were studied. The extract was able to improve endothelium-dependent vasorelaxation in hyperglycemic environments, pointing to its possible vasorelaxant effects.

Hyperglycemia-induced tissue damage and consequent oxidative stress and inflammatory processes, forming a vicious circle, play a pivotal role in the pathogenesis of the vascular complication of T2D [12]. The radical scavenging capacity of anthocyanins has been described in several studies [17,31,32]. Consistent with these studies, the investigated extract has a potent antioxidant effect. However, anthocyanin-rich sour cherry extract at a concentration of 50 ng/µL also significantly reduced ROS compared to the normoglycemia. Increased presence of antioxidants can also result in adverse effects [33]. Therefore, in further experiments, an upper limit should be determined that does not yet cause the phenomenon of antioxidant loading.

To obtain more comprehensive details about the inflammatory processes caused by hyperglycemia, we examined the expression of TNF-α, IL-6, IL-8, and IL-1α. By detecting changes in the gene expression level of our chosen cytokines, the state of the inflammatory profile of cells caused by hyperglycemia can be inferred relatively accurately. While IL-6 is involved in T cell differentiation and B cell stimulation and plays a central role in activating and maintaining inflammatory response, IL-8 is involved in the chemotaxis of neutrophil granulocytes. IL-1α is one of the strongest indicators of immune response to oxidative stress. TNF-α is the central coordinator in mediating cell survival and inflammatory response [34]. Anthocyanin-rich sour cherry extract was able to reduce hyperglycemia-induced overexpression of the investigated genes. Interestingly, lower concentration of anthocyanin-rich sour cherry extract elicited a stronger suppressive effect compared to higher concentration. Although no precise explanation can be given for this, it can be concluded from the literature that some flavonoids are able to activate some receptors at low concentrations, while they exert a desensitizing effect at high concentrations [35]. We assume that increased expression of TNF-α upon treatment at a concentration of 50 ng/µL is the result of this desensitizing effect. To clarify this issue, the signaling pathways of the TNF-α receptor with different concentrations of anthocyanin-rich sour cherry extract should be investigated. Considering our results are limited to PCR assays, further studies are needed in which the signaling pathways of the TNF-α receptor are investigated using Western blot analysis or in-cell ELISA. Moreover, as the extract at a concentration of 50 ng/µL had higher antioxidant capacity but lower immunomodulatory activity compared to 1 ng/µL, the positive effect of anthocyanin-rich sour cherry extract is not only and exclusively due to its antioxidant capacity.

Under hyperglycemic conditions, endothelium-dependent vasorelaxation processes are impaired [36]. One of the main hallmarks of hyperglycemia-induced endothelial dysfunction is the decrease of bioavailable NO [37]. Anthocyanins have been reported to increase the expression of NOS and decrease the expression of various genes responsible for vasoconstriction, including ET-1 [38]. Therefore, we examined the effect of anthocyanin-rich extract on the expression of NO synthase and ET-1 as well as the enzyme producing its active form under hyperglycemic conditions. Our results showed that anthocyanin-rich sour cherry extract was able to improve hyperglycemia-induced damage by enhancing endothelium-dependent vasodilation.

Collectively, in this study, we examined the effect of anthocyanin-rich extract of Hungarian sour cherry on hyperglycemia-induced endothelial dysfunction. The investigated extract has a strong immunomodulatory, antioxidative, and potent vasorelaxant effect. Although further investigations are needed to elucidate the mechanisms of action, our results suggest that Hungarian sour cherry, which is rich in anthocyanins, may have therapeutic potential in diseases associated with endothelial dysfunction, including T2D. Sour cherry can be a component of a diet that supports the prevention and treatment of T2D.
